# Practical Notes on Urinary Analysis

**Published:** 1891-06

**Authors:** 


PRACTICAL NOTES ON URINARY ANALYSIS. By W. B. Canfield, M. D., series of the Physicians' Leisure Library. Published by Geo. S. Davis, Detroit, Mich. Price 25c.

We find nothing new in this little book, though cheerfully recommending it to practitioners as deserving a place among these books of daily references, well illustrated, good paper, close type; the price brings it within reach of every one who desires to know something of urinary analysis. P.

